# Accurate human genome analysis with element avidity sequencing

**DOI:** 10.1186/s12859-025-06191-4

**Published:** 2025-07-25

**Authors:** Andrew Carroll, Alexey Kolesnikov, Daniel E. Cook, Lucas Brambrink, Kelly N. Wiseman, Sophie M. Billings, Semyon Kruglyak, Bryan R. Lajoie, Junhua Zhao, Shawn E. Levy, Cory Y. McLean, Kishwar Shafin, Maria Nattestad, Pi-Chuan Chang

**Affiliations:** 1 Google LLC, Mountain View, CA USA; 2 Element Biosciences, San Diego, CA USA

**Keywords:** Genomics, Sequencing, Bioinformatics, WGS, Variant calling

## Abstract

**Background:**

New sequencing technologies provide options for the scientific community to design studies and build clinical workflows. These options expand user choice, and can enable more accurate, scalable, or affordable workflows depending on the fit between scientist needs and platform capability. However, it is essential to understand the performance of these new technologies for different tasks, especially for capabilities that were not possible or tractable in prior technologies. We investigate the new sequencing technology avidity from Element Biosciences. to help the scientific community understand the performance of the options to generate sequencing data.

**Results:**

We show that Element whole genome sequencing achieves higher mapping and variant calling accuracy compared to Illumina sequencing at the same coverage, with larger differences at lower coverages (20–30x). We quantify base error rates of Element reads, finding lower error rates, especially in homopolymer and tandem repeat regions. We use Element’s ability to generate paired end sequencing with longer insert sizes than typical short–read sequencing. We show that longer insert sizes result in even higher accuracy, with long insert Element sequencing giving more accurate genome analyses at all coverages.

**Conclusions:**

New options for sequencing technologies can analyze genomes comparably or better than prior standard methods.

**Supplementary Information:**

The online version contains supplementary material available at 10.1186/s12859-025-06191-4.

## Introduction

Sequencing the genomes and transcriptomes of organisms enables diagnosis of genetic diseases [1–3], discovery of gene-trait associations [4] for drug discovery [5] and agriculture [6], creation of reference genomes [7], resources for genetic variant annotation [8], and imputation methods [9].

Initially, efforts to assess sequencing accuracy used indirect factors, such as the ratio of transition to transversion in variant calls or concordance with Mendelian inheritance [10]. The ability to assess accuracy was transformed by the Genome in a Bottle standards, a set of 7 human cell lines whose genomes were extensively characterized with multiple technologies, analysis methods, and manual curation [11–15]. This resource, combined with community competitions [16] and comparisons [17] expanded the ability to detect accuracy improvements beyond the accuracy of current individual methods, which allowed a burst of innovation in both sequencing [18] and analysis [19–21] methods to demonstrate the validity of their innovations. This innovation has in turn been shown to increase diagnostic rates and to identify previously missed disease-causing variants [3].

A new sequencing method based on sequencing by avidity rather than sequencing by synthesis developed by Element Biosciences can generate short-read sequencing at high yield, with more than six 30 × genomes in a sequencing run, along with per-base accuracies that reportedly exceed Illumina sequencing [22]. Because the reported metrics focus on the accuracy of individual reads this work assesses how the read level accuracies of Element correspond to accuracy of full genome sequencing including both mapping and variant calling of the Genome in a Bottle samples.

We observe that Element sequencing enables higher accuracy across a range of coverages from 20–50x. The increase in accuracy was most notable at lower coverages (20–30x). We identified genome contexts where Element had improved accuracy, specifically tandem repeats and homopolymers, with a reduction in read soft-clipping due to loss of quality later in the read at these contexts.

One new property of Element’s AVITI platform is the ability to generate paired-end sequencing data with longer insert sizes (the distance between the paired reads) than is typical with Illumina preparations. By investigating Element sequencing runs performed with libraries that had longer insert size distribution (with a template length of > 1000 base pairs as opposed to 350–500 base pairs), we identified a strong positive effect with longer insert sizes for Element sequencing. The long insert Element sequencing outperformed both Illumina and standard insert Element sequencing at each coverage threshold.

## Results

### Comparing variant calling accuracy

We compared genome analysis accuracy between Illumina and Element sequencing in typical use cases. Individual sequencing runs of high sequencing coverage of Illumina [23] and Element were downsampled to equal number of starting reads for 20x, 30x, 40x, and 50 × coverage. These reads were mapped with BWA MEM [24] to the GRCh38 reference [25]. Sequencing runs from HG001, HG002, HG003, and HG005 were analyzed from both technologies. Variants were called with DeepVariant v1.5 [26], which has been jointly trained with both Illumina and Element data in the single release model. All comparisons between technologies use this same DeepVariant model. HG003 is withheld from training all DeepVariant models and that sample is used for whole genome holdout test datasets. Chromosome 20 is withheld from training in all samples and is used for comparison on other samples.

**Fig. 1:**
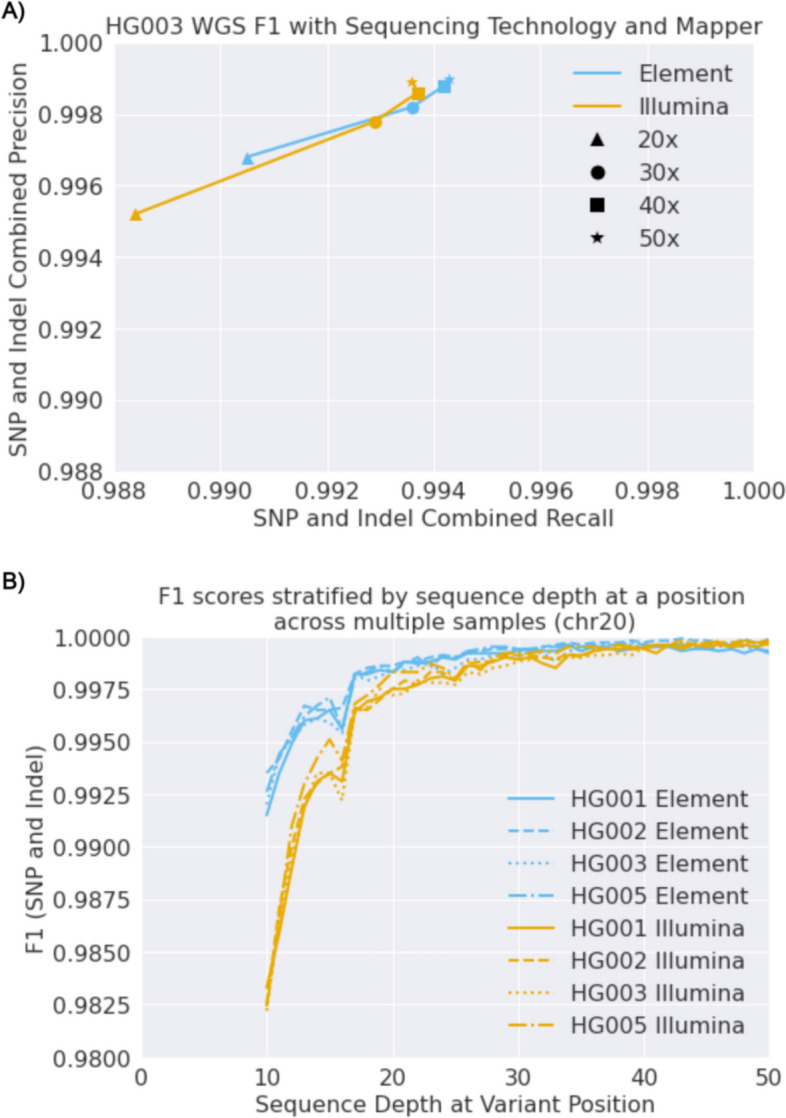
Variant calling accuracy for Element and Illumina sequencing. **A** Variant calling accuracy (precision and recall) with 20–50 × sequencing depth assessed by Genome in a Bottle. **B** Accuracy for Element and Illumina when matching coverage per position between the technologies

To assess accuracy, we used Hap.py [13] to compare the resulting VCF against the v4.2.1 Genome in a Bottle truth set [15] used in the PrecisionFDA v2 Truth Challenge [16]. Element sequencing had higher accuracy (both precision and recall) compared to Illumina at the 20 × coverage point, but the difference narrowed at higher coverage (Fig. 1A, Supplementary Fig. 1).

A sample with 30 × average sequencing coverage will have a distribution of coverages across the genome, for example some positions will be covered at 20 × and others at 40x. To look more directly at the effect of coverage on accuracy for Illumina and Element, we downsampled at 1 × intervals from 50 × to 10x (a total of 40 variant calling runs per sample) across chromosome 20, which is always withheld from DeepVariant training in all samples. We collected the hap.py results for all variant call files, aggregated all calls, and stratified these calls by the sequencing depth at a given position. This allowed us to assess performance on coverage-matched positions across a large coverage range. This revealed larger differences in accuracy at lower coverages between Element and Illumina **(**Fig. 1B**)**. Element had a higher accuracy in the 30–40 × coverage range as well.

**Fig. 2:**
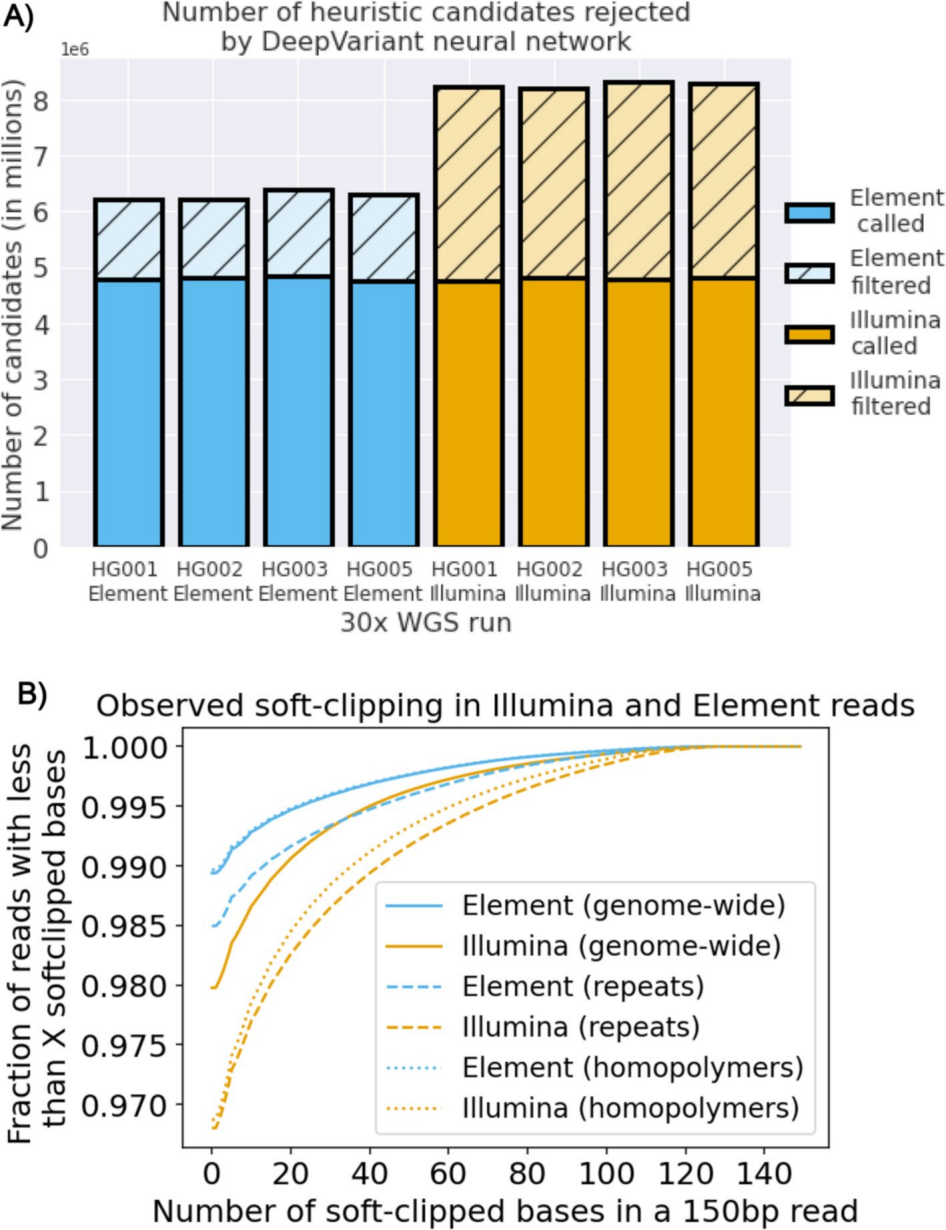
Heuristic approaches which depend on read error rates indicate higher accuracies in Element data. **A** Proportion of candidates proposed by heuristic logic which are rejected as false positives by DeepVariant’s neural net. **B** Proportion of reads with less than a given number of soft-clipped bases for Element (blue) and Illumina (orange) for different reads with MAPQ60 in different regions of the genome. For example, 99% of Element reads in the full genome and homopolymer regions have no soft-clipped bases, while 98% of Illumina reads have no soft-clipped bases genome wide and 97% in homopolymers

The first step of DeepVariant’s variant calling method uses a heuristic process, conceptually similar to Samtools [27] bcftools or GATK [28] which uses observed allele frequencies to propose positions as candidate variants. In the second stage, a convolutional neural network either rejects these candidates as false, or determines they are true and assigns their genotype. In order for a false candidate to be generated, at least two reads must support the candidate and at least 12% of total reads for SNPs or 6% for Indels. At the candidate variant level, we noticed larger differences between Element and Illumina runs. Element runs had fewer rejected candidates (Fig. 2A). The observation of lower false candidate generation was consistent with the reported higher overall accuracy of Element reads [22]. However, the magnitude of the difference seen here should require concentration of errors in certain contexts, as a 12% support rate is much higher than overall Illumina sequencing errors rates. In the case of DeepVariant, the reduction in number of candidates results in a corresponding decrease in runtime for Element relative to Illumina, roughly reducing the DeepVariant runtime by 20%.

The large differences in candidate generation rates between Illumina and Element seemed unlikely to be fully explained by random error rates. Instead, reads going out of sequencing phase in regions difficult to resolve by sequencing by synthesis could generate the error rates required to make candidates. In sequencing by synthesis, reads go out of phase when they hit certain contexts (e.g. homopolymers and tandem repeat runs) that break up the synchronous replication of the cluster, so individual molecules are replicating at different parts of their template [29]. This degrades the sequencing quality and produces errors.

The read mapping step can occasionally identify that a read has useless sequence after a certain point and soft-mask the read. We observed a higher proportion of soft-masked bases in Illumina, which was much more pronounced in repeat regions and homopolymers from the Genome in a Bottle stratifications [30] (Fig. 2B). This is consistent with Element having an improvement in read phasing over difficult contexts.

### Investigating base-level concordance through T2T assemblies

Recently, a highly accurate. telomere-to-telomere (T2T) assembly of chromosome Y was completed for HG002 [31]. This allows us to investigate the empirical accuracy of Element sequencing at the base-level, by taking high quality mapping reads to complete Y-chromosome sequence of HG002 and HG003 and looking for any mismatch from the assembly to create the full base-level error rate of the reads. Because HG003 is the father of HG002 and transmits the same Y-chromosome to the T2T assembly, we can analyze both samples in this way.

**Fig. 3:**
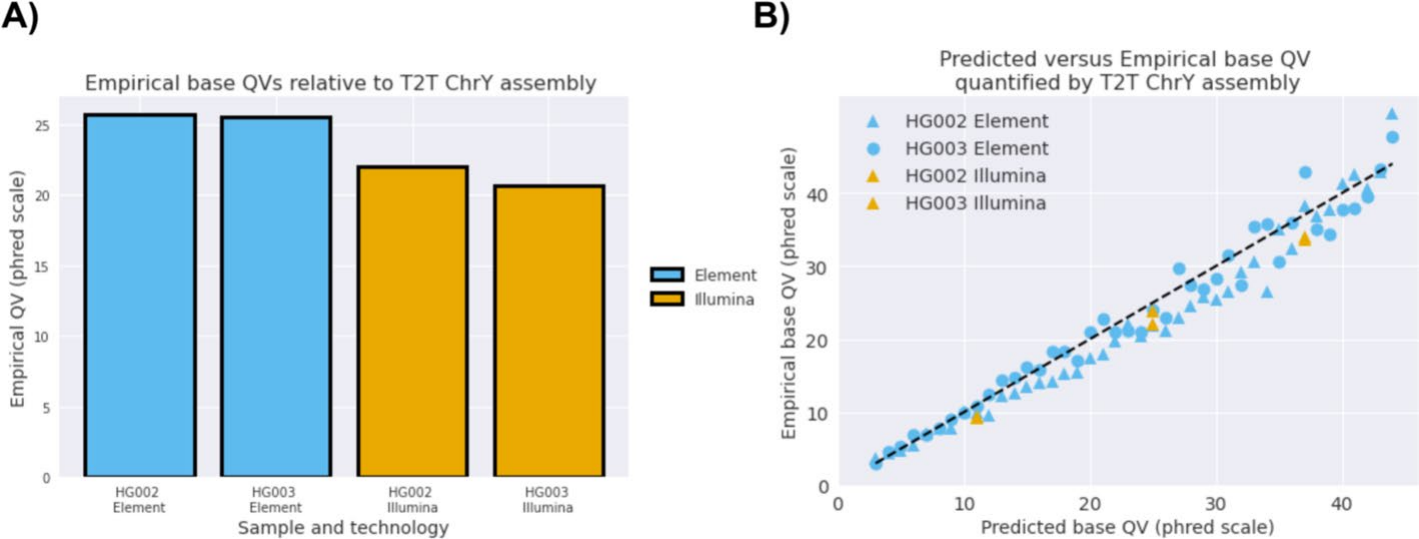
Read accuracy and calibration assessed by concordance with T2T assembly of chromosomeY. **A** Empirical QV for Element and Illumina sequencing runs for two samples (HG002 and HG003). Errors are averaged across all reads mapped to chrY with MAPQ60 in the T2T-XY 2.7 confident regions. The QV is a Phred-scale probability that a sequencing base is correct. Q20 indicates a 99% rate of being correct, Q30 indicates a 99.9% rate of being correct. **B** Calibration between sequencer-predicted base quality (x-axis) with empirical error rate (y-axis). The y = x line represents perfect calibration. The same MAPQ60 reads are used in both comparisons

We used the Bam Error Stats Tool (BEST) [32], which was developed to quantify errors in sequencing technology at the read level by comparing reads to a reliable assembly. Reads at MAPQ60 were used to greatly reduce mapping bias. Consistent with the observations from variant calling, we observe empirical concordance of HG002 and HG003 is higher with Element samples than with Illumina samples. Mismatch rates were 2.4 to 3.3 fold higher in Illumina reads compared to Element (Fig. 3A). We also compare the predicted base quality values and find their calibration consistent between Element samples and well-calibrated beyond predicted Q40 (99.99% accuracy). (Fig. 3B).

### Long insert sequencing improves genome analysis

**Fig. 4:**
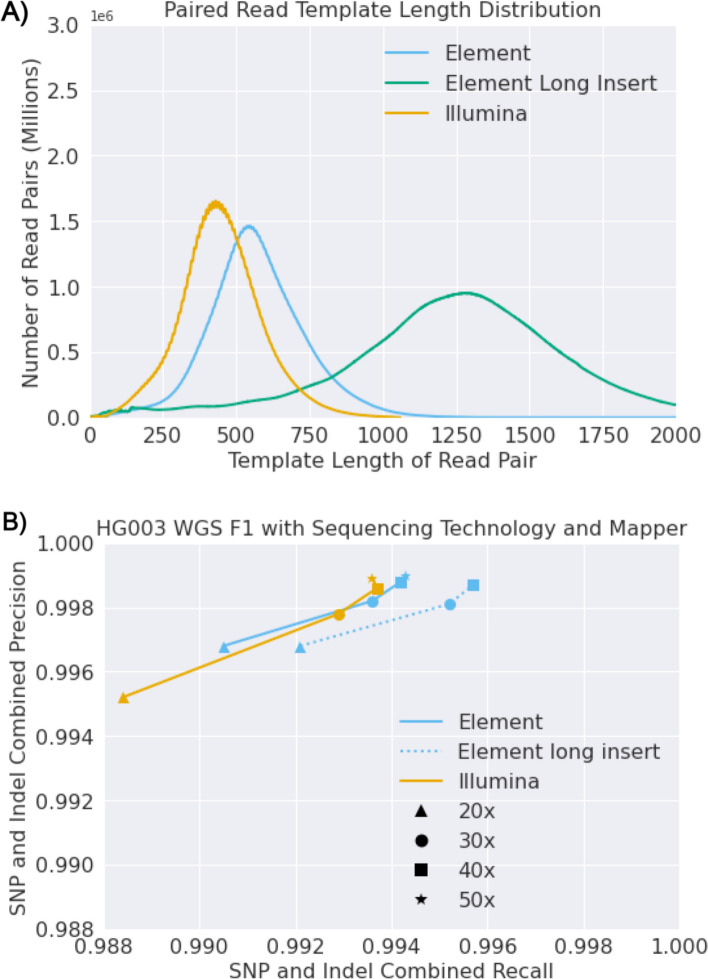
Longer sequencing inserts improve genome analysis accuracy for Element data. **A** distribution of template length for a long insert Element sequencing run versus standard insert Illumina and Element runs. **B** Variant calling accuracy (precision and recall) with 20–50 × sequencing depth assessed by Genome in a Bottle

Element has developed methods that allow libraries with insert sizes of > 1000 base pairs as opposed to 350–500 base pairs to be sequenced efficiently. To test if longer inserts could improve Element sequencing accuracy, we received long insert sequencing runs with a median length of more than 1000 bp (Fig. 4A**)**. The same mapping and variant calling pipeline was used resulting in large improvements in recall (Fig. 4B, Supplementary Fig. 2), suggesting that increasing insert length is a promising mechanism to increase variant calling comprehensiveness and accuracy in general.

In addition to running with DeepVariant, we performed analyses with GATK4.5 on nine additional sequencing runs: 3 Illumina NovaSeq runs, 3 Element runs with the Cloudbreak chemistry and 500 bp insert sizes (standard inserts), and 3 Element runs with the Cloudbreak chemistry and 1000 bp insert sizes (long insert) (Supplementary Fig. 3). Additionally, we express the results for one whole genome sample in total errors (sum of false positives and false negatives for both SNP and Indel errors) (Supplementary Fig. 4). For DeepVariant, we observed the same patterns described previously—Element has higher accuracy than Illumina regardless of coverage, but more pronounced at 20–30x, long insert Element has higher overall accuracy, especially recall. For GATK analyses, we observed that Element had higher accuracy than Illumina at 20 × and 30x, but as coverage increases, GATK gains recall but loses precision. We also observed a trade-off with long insert sequencing which had higher recall for GATK but lower precision. This observation is not present with Illumina. This may suggest that some aspect of the way GATK models the sequencing data was designed for some aspect of Illumina data that is not present in the same way in Element data. DeepVariant at 20 × coverage in every sample, Illumina or Element, achieved a higher accuracy than GATK with any sample or coverage (including 40x). To realize the potential in the long insert Element data, these results suggest that it is ideal to use DeepVariant or a variant caller other than GATK. As with all other analyses, these accuracy measures use regions of the genome never used to train any DeepVariant model (chromosome 20 for all samples) for all samples, and a full sample holdout from training (HG003).

## Discussion

We have characterized the accuracy profile for analysis of human genomes with a new sequencing technology, Element AVITI that uses a sequencing by avidity approach rather than sequencing by synthesis. Element data achieves greater variant calling accuracy over a range of coverages, with especially improved accuracy in the 20–30 × coverage range. We identify certain sequence contexts in which Element outperforms Illumina reads, including in tandem repeats and homopolymers, as measured by soft-clipping rates. Finally, we show a positive effect on the accuracy of whole genome sequencing pipelines when using longer inserts for sequencing.

Although these investigations focus on whole genome analysis for germline variation at coverage ranges typically used for variant discovery, there are several other applications for which base-level accuracy is of greater importance. These applications include somatic sequencing for detection of subclonal acquired variants, deep sequencing of cancers, or analysis of cell-free DNA. For this application, only a few sequence reads may contain a variant at low allele fraction, and the ability to determine whether those bases reflect a real variant or an error depends highly on sequence quality. Similarly, low-pass sequencing of samples followed by imputation could benefit more, which has recently been investigated with Element sequencing [33].

The high accuracy of Element in homopolymers and repeats could provide a unique ability to improve genome assemblies and reference resources by polishing remaining errors in these contexts which are difficult both for Illumina as well as long-read methods like Pacific Biosciences and Oxford Nanopore.

One caveat in analyses of accuracy is that the current (v4.2.1) Genome in a Bottle benchmarks do not cover the entirety of the Genome, due to the difficulty in mapping certain parts of it. Accuracy across the full genome, including these parts not covered by Genome in a Bottle is likely lower. The longer insert size Element runs might be able to better access parts of the genome which can’t be measured by these benchmarks, and could be another method to help expand the confident regions in future releases.

## Conclusions

We demonstrate that data from the new short-read sequencing instrument Element AVITI can achieve comparable or better performance compared to Illumina NovaSeq. This will expand the available options for the research community for sequencing technology choice.

We identify areas of better performance, including higher accuracy especially at lower coverage, and a reduced amount of read soft-clipping in repetitive regions and homopolymers. This will allow the research community to better resolve certain challenging genomics regions.

We demonstrate that the use of longer inserts between read pairs when sequencing can improve accuracy, especially recall, which we hope will shift the research community and technology providers toward using longer inserts to improve mappability and analysis of genomes.

## Methods

### Protocol for long insert element data

Covaris-sheared, PCR-free long insert libraries were prepared using the Kapa HyperPrep workflow. 1ug of HG002 and HG003 gDNA were mechanically sheared using the following Covaris program:

**Table:** 

Duration	Temp	Peak power	Duty % factor	Cycles/bursts	Average Power
10 s	12C	50	20	200	10

A narrow double-sided SPRI selection ratio of 0.3X/0.42X was used to select the long fragments. The Adept Rapid protocol was used for circularization. The libraries were sequenced on the Element AVITI system, 2 × 150 paired end reads with indexing, using a custom recipe for long inserts. The primary changes to the recipe involved increasing the amplification time to account for the increased insert length.

### Reference genome used

GRCh38 with masking of certain false segmental duplications as recommended by Genome in a Bottle [34] (GRCh38_masked_v2_decoy_excludes_GPRIN2_DUSP22_FANCD2.fasta.gz) was used for all germline variant calling pipelines. For BEST analysis ChrY of T2T-CHM13v2.0 [31] was used.

### Read mapping

Mapping was performed with BWA v0.7.17 (r1188) [24]. Duplicate marking was performed with GATK v4.1.2 [28].
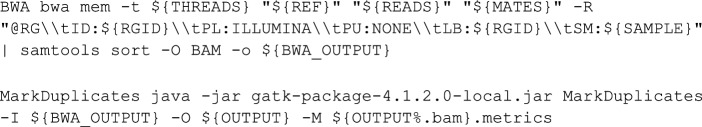


### Variant Calling

Variant calling was performed with DeepVariant v1.5 [26] using the WGS model.
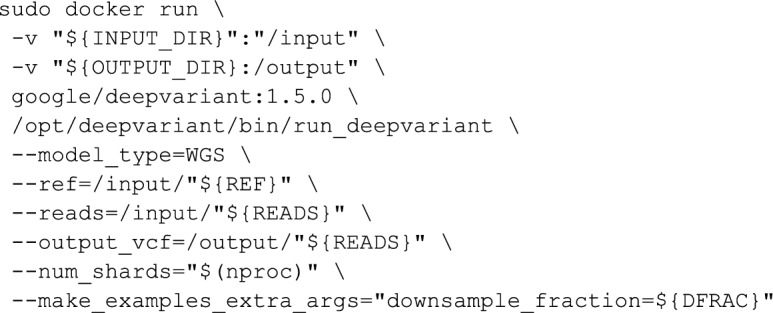


### Read and base level assessment

Assessment of base and read level accuracy was performed with BAM Error Stats Tool (BEST). [32]
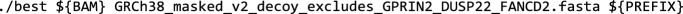


Assessment used only MAPQ 60 reads in the T2T-XY v2.7 confident regions. The confident BED file used is at: https://storage.googleapis.com/brain-genomics-public/research/element/chry/T2T_chrY_confident.bed

### Variant accuracy evaluation

Accuracy evaluation was performed with hap.py [13] using the v4.2.1 [13, 15] truth sets from Genome in a Bottle.
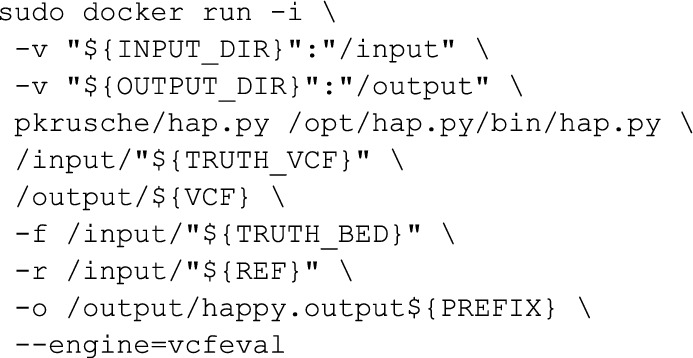


### Chromosome20 downsampling

To assess accuracy matching coverage at variant position for Fig. 1B, downsamples at 1 × intervals were conducted from 50 × to 10x, and hap.py used to annotate variant calls as true positives, false positives, and false negatives. All calls for a given sample were aggregated over the files, and the sequence depth for each variant position was used to calculate total precision and recall.

### Read count and downsampling fraction for sequence data

The sequencing data used for this paper is available as FASTQ files at: https://console.cloud.google.com/storage/browser/brain-genomics-public/research/element/sequencing_files/

The number of reads for each sample (including both paired files) and the downsample fraction required to reach 30 × coverage are:

Downsampling is performed by generating a new BAM with the command:
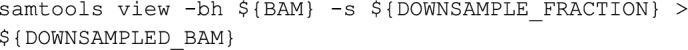



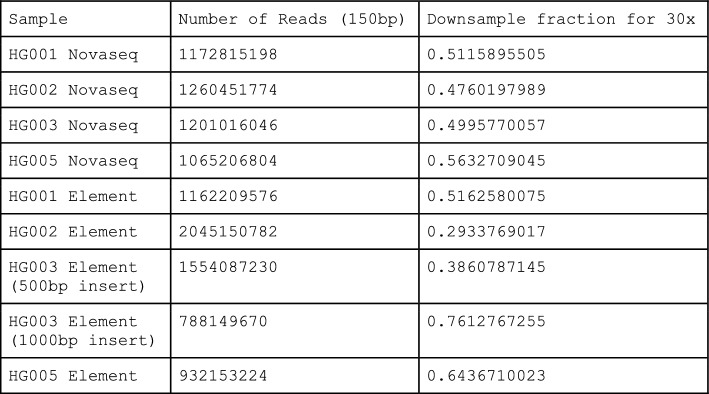


## Supplementary Information

Supplementary Material 1.

## Data Availability

Illumina sequencing was taken from PCR-Free NovaSeq6000 data generated as described in Baid et al. [23]. Element sequencing data for whole genome comparison on HG003 was taken from the Cloudbreak release. Sequencing data from other samples was taken from earlier Element chemistries and made available by Element from: https://www.elementbiosciences.com/resources. FASTQ, BAM, VCFs, and analysis files are hosted publicly and available with no egress charge at: https://console.cloud.google.com/storage/browser/brain-genomics-public/research/element/. Accessible from GCP console at: gs://brain-genomics-public/research/element/. All contents of this folder are available via direct https links, an index of file urls can be downloaded at: https://storage.googleapis.com/brain-genomics-public/research/element/element_urls.txt. Within this folder there are five subfolders: candidates/—VCF files for 30 × sequencing of Illumina and Element multiple samples used to identify filtered candidates across samples (Fig. 2A). chr20/—Chromosome20 VCF files for 1 × downsamples from 10 to 50 × of Illumina and Element samples. Used for Fig. 1B. chry/—ChromosomeY BAM, VCF, and Best analysis files used to assess read concordance with T2T assembly. Used for Fig. 3A–B. sequencing_files/—Whole genome sequencing FASTQ files analyzed in this paper. wgs/—FASTQ, BAM, VCF, and Hap.py files for HG003 Illumina and Element Cloudbreak, include standard (500 bp) and long (1000 bp) insert sizes. Used for Figs. 1A, 2B, 4A–B.

## Abbreviations

BAM: Binary alignment map
VCF: Variant call format
T2T: Telomere-2-telomere
GRCh38: Genome reference consortium human build 38
BEST: Bam error stats tool
WGS: Whole genome sequencing
GCP: Google cloud platform
NIST: National institute of standards and technology
