# Supplementary material for: Accurate human genome analysis with element avidity sequencing
Source: BMC Bioinformatics. 2025 Jul 25;26:194. doi: 10.1186/s12859-025-06191-4 (PMC12291380; doi:10.1186/s12859-025-06191-4)
Supplement: Supplementary file 1 — Supplementary Material 1. [file 12859_2025_6191_MOESM1_ESM.docx]

### Supplementary Figures

**A)**

**
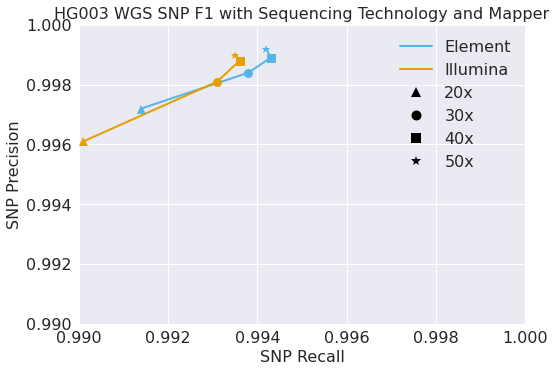
**

**B)**

**
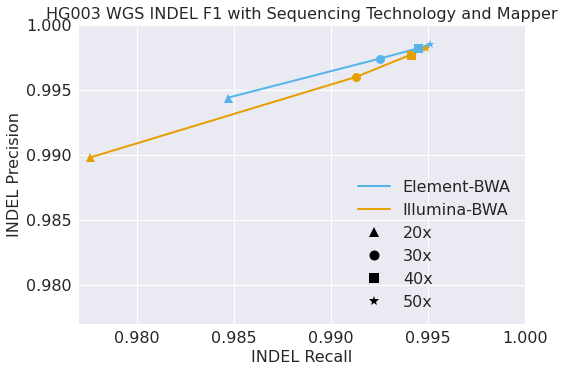
**

**Supplementary Figure 1**. *Variant calling accuracy for Element and Illumina sequencing separating SNP and Indels.* **(A)** SNP Variant calling accuracy (precision and recall) with 20x-50x sequencing depth assessed by Genome in a Bottle. **(B)** Indel Variant calling accuracy (precision and recall) with 20x-50x sequencing depth assessed by Genome in a Bottle.

**A)**

**
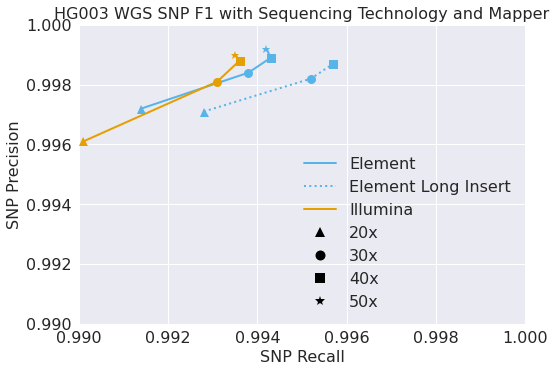
**

**B)**


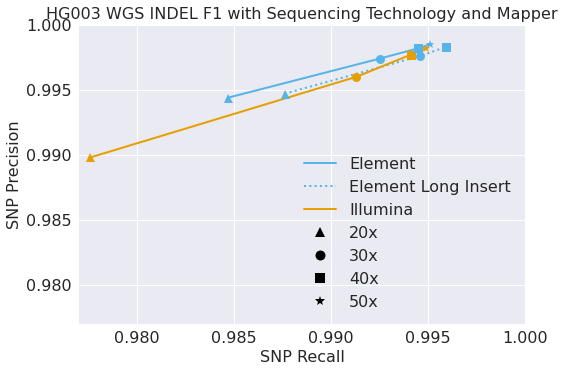


**Supplementary Figure 2**. *Longer sequencing inserts improve genome analysis accuracy for Element data separating SNPs and Indels.*  **(A)** SNP Variant calling accuracy (precision and recall) with 20x-50x sequencing depth assessed by Genome in a Bottle. **(B)** Indel Variant calling accuracy (precision and recall) with 20x-50x sequencing depth assessed by Genome in a Bottle.


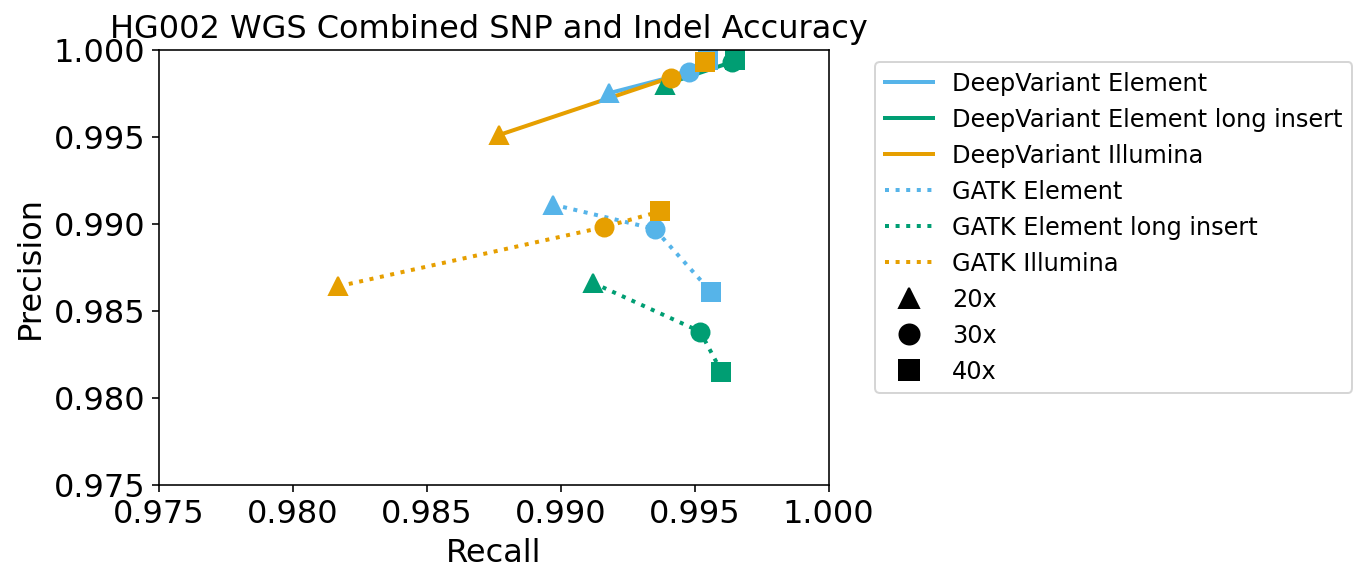


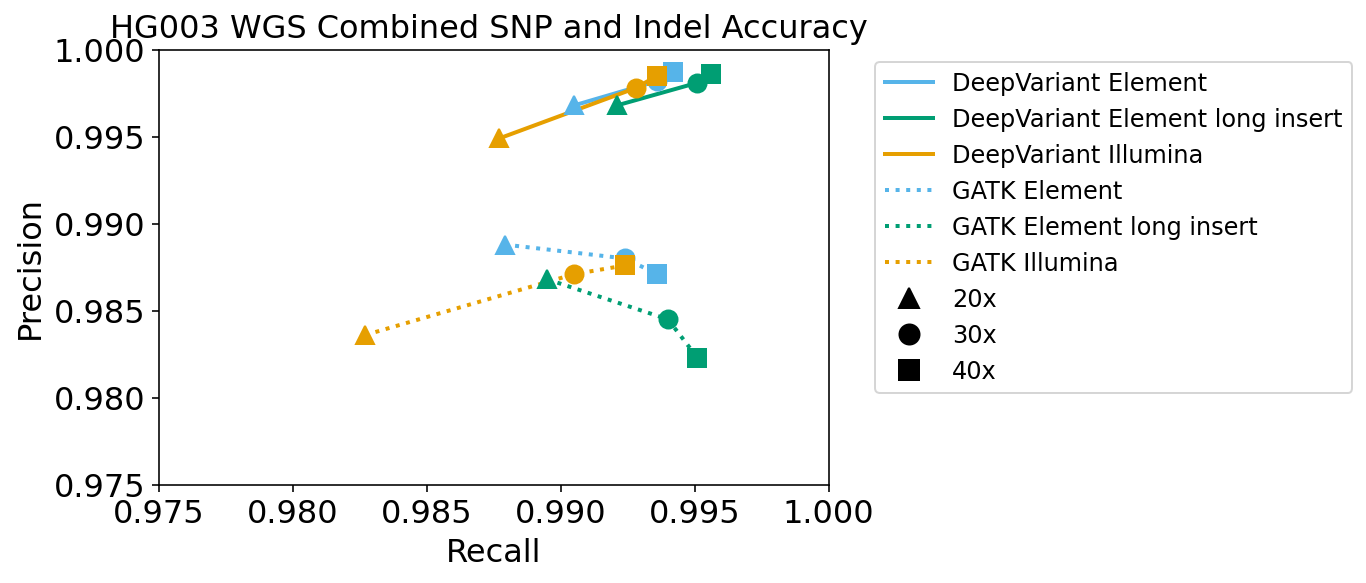


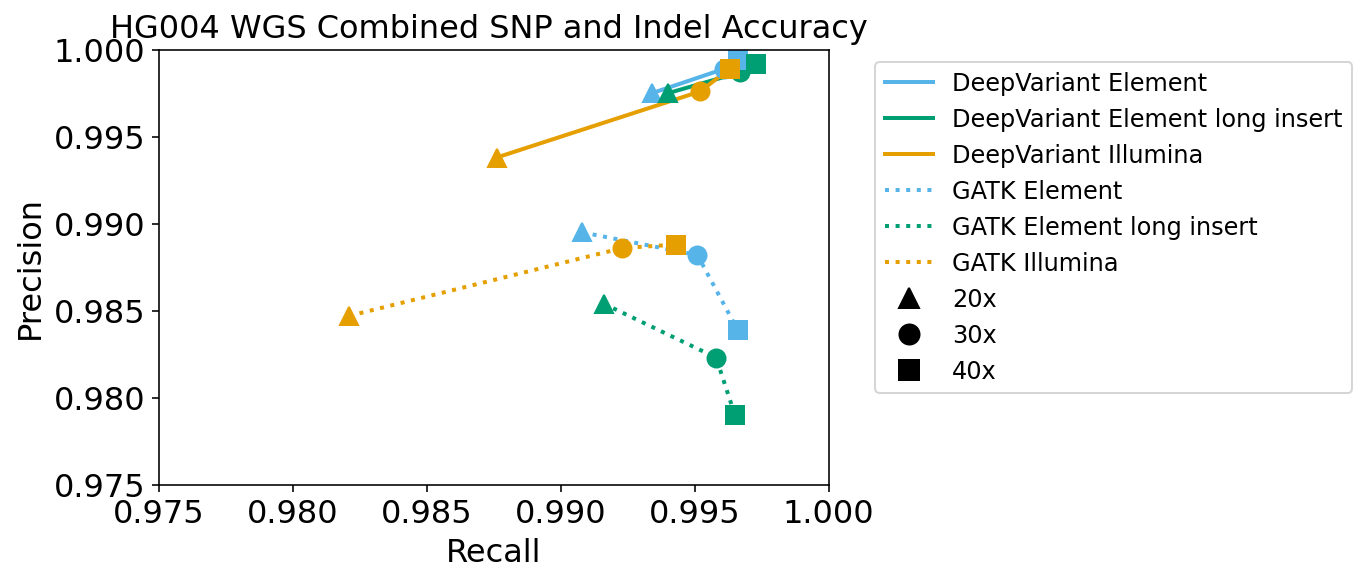


**Supplementary Figure 3**. *Comparisons between Illumina and Element standard and long insert analyzed with both DeepVariant and GATK*. For three samples: **(A)** HG002, **(B)** HG003, **(C)** HG004 at 20x, 30x, and 40x sequencing depth assessed by Genome in a Bottle. Analyses with DeepVariant are shown in solid lines and with GATK in dotted lines.


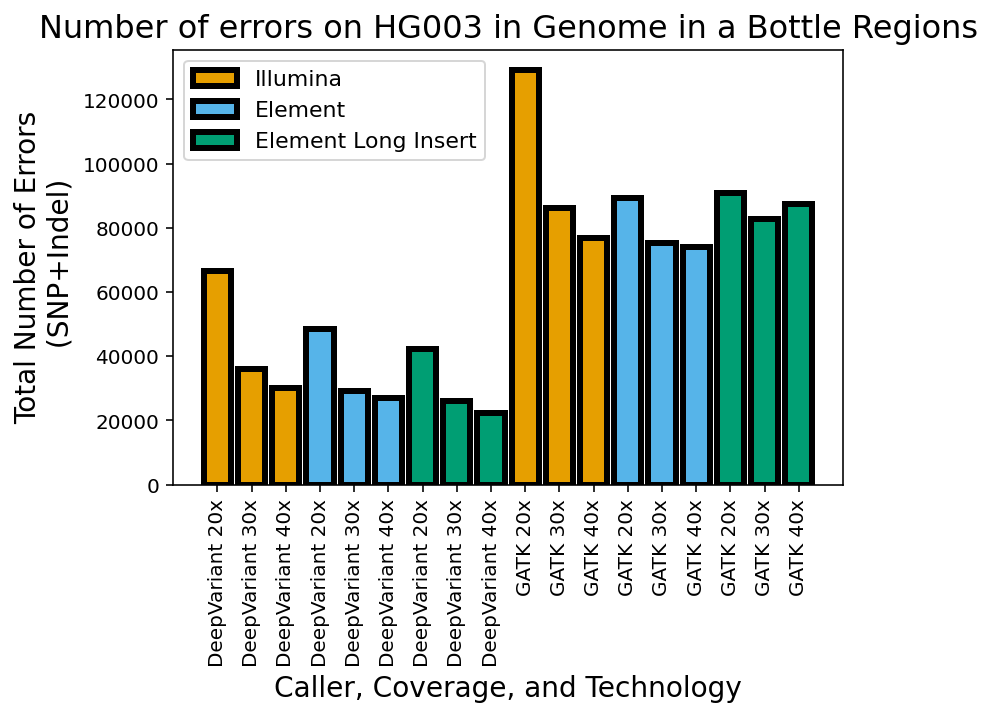


**Supplementary Figure 4.** *Total errors with Illumina, Element, and Long-Insert Element data analyzed with both DeepVariant and GATK at different coverages****.*** Errors are the combination of false negatives and false positives summed for both SNP and Indels across the Genome in a Bottle regions v4.2.1.
